# Ethical Challenges in Oral Healthcare Services Provided by Non-Governmental Organizations for Refugees in Germany

**DOI:** 10.1007/s11673-023-10327-7

**Published:** 2024-02-14

**Authors:** R. Kozman, K. M. Mussie, B. Elger, I. Wienand, F. Jotterand

**Affiliations:** 1 Institute for Biomedical Ethics, University of Basel, 4056 Basel, Switzerland; 2 Medical College of Wisconsin, Milwaukee, Wisconsin USA; 3 University of Basel, Basel, Switzerland

**Keywords:** Dental public health, Oral health access, Health equity, NGO, Germany

## Abstract

Oral healthcare is attracting much attention after decades of neglect from policymakers. Recent studies have shown a strong association between oral and overall health, which can lead to serious health problems. Availability of oral healthcare services is an essential part of ensuring universal healthcare coverage. More importantly, current gaps in its accessibility by minority or marginalized population groups are crucial public health as well as ethical concerns. One notable effort to address this issue comes from Non-Governmental Organizations (NGOs), which offer oral healthcare services for non-insured refugees. However, the challenge remains that these care services are not comprehensive, which has implications for the refugees’ oral and general health. In this article, we discuss this complex issue in the German healthcare context by including ethical reflections. Therefore, the purpose of this article is to discuss the ethical challenges related to oral healthcare services provided by NGOs for refugees in Germany. First, we will introduce the general oral healthcare context worldwide and in Germany. Second, we will provide a general description of the oral healthcare services provided by NGOs for refugees in Germany, as well as an overview of existing gaps. This will provide us with the context for our third and most important task—discussing the ethical implications of the gaps. In doing so, and since the ethical implications can be several, we demarcate the scope of our analysis by focusing on the specific ethical issues of justice, harm, and autonomy. Finally, we offer some recommendations for how to move forward.

## Introduction

Oral diseases are growing global health concerns affecting more than 3.5 billion people worldwide (World Health Organization 2022). According to the Global Burden of Disease (GBD) in 2019, untreated dental caries in permanent teeth was the most common disease condition (Vos, et al. 2020). Adding to this burden is the limited and unaffordable availability of oral healthcare services regardless of how developed is the given healthcare system. Despite the vital role of oral health services in promoting general health and quality of life, evidence shows that they are unaffordable for more than four billion people globally (Naavaal, Griffin, and Jones 2020). Socioeconomic inequalities influence the variation in access to oral healthcare from one country to another (Winkelmann, et al. 2022). Regardless, the lack of access to oral healthcare may lead to severe oral health consequences and systemic health complications (American Dental Association 2023). Efforts to address this challenge remains limited despite oral diseases being mostly preventable global public health challenges and having serious health consequences. This gap is a serious public health and ethical issue, especially when considering the premise that the provision of equitable healthcare is one of the vital means towards attaining human flourishing and justice (Jotterand, et al. 2022). Although justice is a complex concept that goes beyond the scope of this paper, the limited accessibility and affordability of oral health services discussed here align with the concept of justice in healthcare, which aims to ensure a health system that benefits everyone (Habibzadeh, Jasemi, and Hosseinzadegan 2021; Lee and Divaris 2014).

Oral health diseases and the inaccessibility of oral healthcare services are serious challenges among refugees in Europe in general and in Germany in particular (Bhusari, et al. 2020; Zinah and Al-Ibrahim 2021; Goetz, Winkelmann, and Steinhäuser 2018; Al-Ani, et al. 2021). We applied the definition of refugee given by the United Nations High Commissioner for Refugees (UNHCR), which is referred to as “people who have fled war, violence, conflict or persecution and have crossed an international border to find safety in another country” (UNHCR 2023, ¶1). The United Nations High Commissioner for Refugees reported that by the end of 2019, 79.5 million people had been forcibly displaced globally due to a variety of events such as wars, human rights violations, or other similar factors (UNHCR 2019). In 2020, more than 3.3 million individuals were forcibly displaced to several European countries such as Germany, Spain, Italy, and France. Germany received the most of non-EU refugees than any other European country (Eurostat 2022). Oral health challenges and the limited availability of adequate oral healthcare among such a growing population group imply public health as well as ethical concerns of, among others, distributive justice and autonomy in the healthcare of refugees (Straehle 2020).

Lack of access to healthcare being a serious ethical issue per se, limited availability of oral healthcare services among refugees, especially among those whose refugee status is not yet accepted, poses matters of fairness and justice (Bhusari, et al. 2020; Cribb, Entwistle, and Mitchell 2020; Mussie, et al. 2022) In Germany, one cross-sectional study reports “high prevalence of untreated caries and poor oral hygiene among newly arrived refugees” (Solyman and Schmidt-Westhausen 2018, 1). Similarly, a national oral health survey among refugees reports that “refugees had high caries experience, often untreated caries teeth and more complications compared with the German resident population” (Al-Ani, et al. 2021, 2399). The definition of restricted healthcare access according to §4 Asylum-Seekers’ Benefits Act (AsylBLG) prohibits refugees from getting oral health treatment unless they experience pain, and this practice of postponing treatment until there is pain is not preventive (AsylbLG 2022). This leads to the discrimination of refugees’ healthcare and causes higher costs for both refugee patients and the health system (Goetz, Winkelmann, and Steinhäuser 2018; Spinler, et al. 2022). To mitigate these challenges, NGOs engage in addressing the oral healthcare needs of refugees across Germany (Gamarra, et al. 2021).

The aim of this paper is to identify the gaps and ethical implications of oral healthcare services for refugees in NGO contexts in Germany. The reasons behind choosing this topic are: (a) to contribute to deliberations on ethical challenges in oral healthcare, (b) to integrate the in-depth experience of the first author RK as a dentist working with refugees in Germany, and (c) because this topic is under-researched although refugee health is a serious public health concern in Germany that necessitates urgent attention (Goetz, Winkelmann, and Steinhäuser 2018; Al-Ani et al. 2021). In discussing this topic, first, we start by providing a summary of the general and oral healthcare coverage for refugees in Germany. Second, we give a summary of the NGO context and present the challenges with their ethical implications. Finally, we provide concluding remarks and a few recommendations. In this article, our arguments are supported by examples from different contexts as current evidence on the topic is highly limited. Moreover, a note of caution is due as our description is not comprehensive but only mirrors our multidisciplinary perspectives on ethical issues as we come from backgrounds such as dentistry, bioethics, public health, and philosophy.

## Oral Healthcare For Refugees In Germany and the Insurance Context

Medical and dental care provided to refugees in European countries exhibit notable distinctions in terms of their scope, providers, accessibility, coverage, specialized considerations, integration within healthcare systems, and healthcare policies. Dental care primarily focuses on oral health services, while medical care encompasses a broader spectrum of healthcare services, potentially including primary care, specialty care, and emergency medical services. The provision of dental care for refugees may face challenges in terms of availability, waiting times, and limited coverage compared to medical care, which is more deeply integrated into the overall healthcare system of host countries. Furthermore, refugees may present with specific dental needs due to previous experiences, necessitating tailored dental interventions. These variations arise from the diverse healthcare policies and resource allocations of individual European countries and their evolving approaches to refugee healthcare.

In Germany, there are differences between insured and non-insured individuals or refugees in terms of accessibility and out-of-pocket payments for oral healthcare. Citizens are obliged to be health insured by the German social security law, which mostly enables them to receive healthcare treatments anywhere (hospitals and clinics) and at any time free of charge (Federal Ministry of Health 2020). Insurance companies and healthcare facilities work together to minimize out-of-pocket expenditures for insured citizens and provide a full dental treatment plan (Bock, et al. 2014). A full dental treatment plan mainly consists of two phases: an acute pain management phase where the aim is to simply relieve pain through procedures such as caries removal and root-canal treatment, and a restorative phase where the focus is preserving and restoring the functionality that might have been lost after performing the acute treatment phase (Medscape 2021). Thus, such full dental treatments are facilitated for insured citizens using measures such as, for example, a fixed subsidy of 60 per cent for standard treatment of crowns or onlays (Winkelmann, et al. 2022). The same applies to insured citizens in France, the United Kingdom and most of the European countries where patients are free to choose between statutory and private dentists where a compulsory out-of-pocket payment would be needed in order to perform specific restorative and aesthetic treatments (Winkelmann, et al. 2022; Mazevet, et al. 2018).

On the other hand, refugees in Germany do not have the same insurance privilege as the insured citizens. For example, in the first eighteen months of the asylum-seeking period, the AsylbLG limits the asylum seeker from accessing all healthcare services that other patients in the statutory health insurance (GKV) have access to. Elaborating on this challenge, a hospital-based retrospective study in Germany reports that “the legal situation entails a number of administrative barriers that make access to dental care more difficult for asylum-seekers compared to regularly insured patients” and this makes utilization of dental care among the non-insured lower than among the insured (Freiberg, et al. 2020, 8). Thus, such population groups who are not included in the insurance system mainly seek free dental treatments from NGO facilities (Freiberg, et al. 2020; Pichemin, et al. 2022).

## NGOs Addressing the Refugee Oral Healthcare Gaps—A Synopsis of Opportunities and Challenges

In recent years, and especially in 2015, mass displacement and population migration have greatly compromised healthcare provision for refugees and undocumented migrants in Germany. According to one qualitative study reporting on the engagement of NGOs to address this challenge, this crisis “exposed regulatory and structural shortcomings with respect to refugee healthcare provision” (Brenner and Lok 2022, 1). On top of health access being limited by the AsylbLG, several municipalities experienced administrative and logistic constraints to even provide critical healthcare services that the immigration laws already grant refugees (Bozorgmehr, et al. 2016; Wahedi, Nöst, and Bozorgmehr 2017). There was increased involvement from non-governmental bodies to address these challenges. Both international and national humanitarian organizations collaborated with the government, civil societies, the private sector and other stakeholders to mitigate the situation in Germany together with several other European countries that were affected by the refugee crisis (Papuc 2017).

NGO engagement with healthcare provision for refugees increased significantly after the 2015 refugee crisis (Brenner and Lok 2022). Their contributions included “collaborating or supporting their municipalities in planning and implementing publicly coordinated efforts to organize and provide healthcare provision in these camps” (Brenner and Lok 2022, 6). Part of these efforts from NGOs also included providing coverage for non-insured (by the state social welfare system) healthcare services such as dental care among the population group. For a non-insured or refugee patient, an out-of-pocket payment is often required in order to receive dental treatment (Wahedi, Nöst, and Bozorgmehr 2017). However, due to the social and financial state of the refugee patients, out-of-pocket payments are mostly not feasible. Therefore, the patients are obliged to seek free treatment, which is in this case offered by NGOs which provide such treatment without requiring co-payment by the patient or asking for minimal sums depending on what the patient can afford.

NGOs lack the financial and human resources needed to offer patients a comprehensive dental treatment plan, further affecting the quality of care refugee patients receive (Freiberg, et al. 2020). The World Health Organization (WHO) defines quality of care as “the degree to which health services for individuals and populations increase the likelihood of desired health outcomes” (World Health Organization 2023, ¶1). NGOs operate with conservative operational costs with a limited number of volunteers such as physicians, nurses, and co-workers. This shortage of human resources highly affects the sustainability and comprehensiveness of the treatments provided for refugee patients, thereby affecting the desired outcomes (Mowafi, et al. 2007). Thus, contact hours with patients are kept short to accommodate a high number of patients in a limited time, which implies the risk of missing important patient information and overlooking the comprehensive treatment plan of the patient.

In addition, and most importantly, NGOs do not offer all treatment options such as dental filling, periodontal, and root canal treatment because such treatments require multiple sessions and could be costly. Such a lack of comprehensive treatment can have implications on both oral as well as general health (Gordon, Mosen, and Banegas 2021). In support of this, a recent study reporting on the association between deteriorated oral health status (periodontitis) and general health conditions in Latin America highlights that patients suffering from periodontitis are also facing negative systemic worsening of their general health and other clinical conditions (Fischer, et al. 2020). Additionally, other studies have shown that periodontitis possibly worsens the health condition of patients suffering from diabetes, kidney dialysis, gastrointestinal, and cardiovascular diseases (Preshaw and Bissett 2019; Miyata, et al. 2019; Bao, et al. 2022; Carrizales-Sepúlveda, et al. 2018). Such negative health consequences of incomprehensive oral healthcare are captured in a study conducted among Syrian refugees in Jordan (Salim, et al. 2021).

## Ethical Implications

Without disregarding the oral health benefits refugees get due to NGO involvement, there are still gaps in healthcare delivery that warrant ethical deliberation. In what follows below, we will divide the discussions into two parts. First, we discuss the implications of the challenges for the principle of distributive justice. Here, we also relate justice with harm by giving examples of the unfavourable consequences of some treatment practices. Second, we show how decision-making processes can conflict with the principle of autonomy.

### Injustice and Harm

The oral health services refugee patients receive from NGOs could be described as lacking the needed quality in comparison with services provided for the general population. This gap, especially when it concerns vulnerable population groups, poses an ethical concern as it could imply a systemic injustice (Wibowo, et al. 2019). Evidence shows that NGOs providing healthcare (in general) for refugees in Germany have resource challenges such as manpower and budget, thereby affecting the health services they offer their refugee patients (Brenner and Lok 2022; Kratzsch, et al. 2022). For example, and as established in earlier sections, oral health treatments from NGOs mostly cover one aspect (acute emergency treatment) of the treatment and do not provide comprehensive care. This implies that the refugee patient could experience further oral and general health complications due to a healthcare treatment procedure that could have been done otherwise and better regardless of whether improving the care is beyond the resource capacity of the NGOs. On the contrary, a comprehensive dental treatment would be offered to the remaining population. A broad discussion of why the NGOs are experiencing financial challenges and how that affects the oral healthcare services for refugees warrants further investigation and falls beyond the scope of this paper.

The health insurance conditions for refugees in Germany could further demonstrate the gaps in the provision of healthcare. According to the AsylbLG, a refugee’s insurance status changes from “non-insured” to “insured” after the first eighteen months. Thus, patients who received their dental treatment at the NGO when they have the “uninsured” status would have a future change in their health insurance status and that gives them the right to receive a treatment, which will be covered by the statutory insurance (Wenner, et al. 2022). Although this is a good approach per se, it illuminates the (oral) health complications refugees experience during their “uninsured” status. As a result of the previous non-comprehensive dental treatment at the NGO facility, the clinical condition of the patient drastically deteriorates after not receiving the comprehensive treatment plan during the first 18 months. When the patient shows up for dental treatment at this future point with the expected deteriorated clinical condition, the statutory insurance is, therefore, obliged to cover the expenses of any clinical condition and any restorative need that was not offered by the NGOs when the refugee had an “uninsured” status (Bozorgmehr, et al. 2016).

Untreated health complications also do injustice to health system. Offering suitable comprehensive treatment to the “uninsured” refugee patient through NGOs would have been more cost-effective than only offering emergency care and leaving the remaining clinical concerns. Treating both dental and systemic complications would be a double burden for statutory insurance as insurance firms have to cover expenses for restorative and the treatment of systemic complications. This is a complex situation for both refugee patients and health systems. Moreover, this gap shows the lack of ensuring Universal Health Coverage (UHC)—a principle that the WHO considers as a powerful tool to achieving fairness and solidarity in healthcare provision (United Nations 2023, ¶1). Achieving social justice in healthcare, regardless of whether it is oral health or other types, is in line with the principles of UHC (Pande, El Shalakani, and Hamed 2017). Upholding UHC as a principle is highly beneficial in achieving fairness in healthcare provision. Ensuring justice in oral healthcare is, more broadly, critical in addressing systemic inequalities and ensuring that all individuals have access to essential healthcare services.

## Lack of Autonomy

Patient autonomy is considered a crucial concept in medical decision-making. With the help of the treating physician, it is the right of every patient to decide which treatment is the best for their condition (Iserson 1999). Lack of this choice means that the decision of a patient would highly be affected by external influences, meaning decisions are constrained by others’ plans and patients cannot decide independently (Iserson 1999; Entwistle, et al. 2010). Most autonomy theories analyse these issues considering two criteria: independence from external influences, and the capacity to make intentional choices and act according to these choices (Pugh and Pugh 2020). The idea behind autonomy is informing the patient about the available treatment options that are suitable for them and allowing the patient to choose freely without influencing that decision. Indeed, this is without disregarding the importance of providing all the needed information regarding treatment options and their possible consequences (Jotterand, Amodio, and Elger 2016).

Due to the lack of affordability of various dental treatments offered by the NGO, patients may feel compelled to accept acute emergency treatment as their only available option. This situation highlights a previously unseen and neglected aspect of autonomy in dental care, wherein external financial pressures play a significant role. This circumstance raises ethical concerns regarding the impact of financial barriers on patient autonomy. When patients are financially coerced into accepting a specific treatment due to its affordability, their autonomy becomes compromised. Ethical principles stress that treatment decisions should be based on patient preferences and medical considerations, rather than external financial constraints. In essence, this situation suggests that patients may not have the freedom to make decisions independent of external circumstances, which can undermine their autonomy. This aspect of autonomy may often go unnoticed and unaddressed within dental treatment settings operated by NGOs. It underscores the importance of healthcare providers and organizations being vigilant in upholding patient autonomy, even in resource-constrained environments. Furthermore, there is an additional ethical concern related to the potential harm experienced by refugee patients who receive only acute emergency dental care. The paragraph suggests that such patients may encounter clinical complications that can ultimately impact their overall health. This raises important questions about the ethical responsibility of healthcare providers and organizations in delivering comprehensive care that considers both short-term and long-term consequences for patients, highlighting a potential violation of patients’ rights.

Further elaborating on the possible lack of autonomy refugee dental patients could experience, a related ethical issue that arises if a lack of informed consent. Dental treatment is advancing and incorporating several treatment options for patients. In the past, dental care options were limited compared to today’s modern dentistry, which offers a wider range of treatment options (Kaur and Singh 2018). Nowadays, modern dentistry offers a full scale of treatment options, which require the patient to provide written informed consent. The bioethical principle of autonomy, at least in its traditional essence, can be considered respected when the patient is informed about the whole treatment modalities and provides (verbal or written) consent (Entwistle, et al. 2010). In the case of written informed consent, the form that is used is a legal document which asserts the right of the patient and documents the consent or agreement upon the treatment that is about to begin.

Implementing informed consent in the NGO setting and informing each patient about the whole treatment course in different languages is very time and resource-consuming. In most humanitarian settings, due to time pressure and human resources deficiency, it is very challenging to ensure informed consent implementation as the urgent focus is pain alleviation (Hussein and Elmusharaf 2019). Thus, despite lack of informed consent being a serious ethical issue in healthcare in general, NGOs tend to hand consent forms to refugee patients only after extraction is performed and as a means of instructing the refugee patients concerning what to do after the extraction (from first author’s experience). Furthermore, the availability of other templates and informed consent forms for other treatment options is lacking. Therefore, explaining the treatment aspects is not well performed as each case clinically differs from the other. This is a serious ethical matter since patients mentally capable of comprehending the treatment plan are entering a treatment without getting full information and providing a formal agreement.

The communication gap between patients and physicians is a significant challenge in ensuring patient autonomy, particularly among refugees receiving oral healthcare in NGO settings in Germany. More specifically, the language barrier is a critical factor that impedes patients' ability to understand and consent to the proposed treatment plan. To establish an effective patient–physician relationship and provide quality healthcare, physicians must be understood and perceived as trustworthy by patients (Mussie, et al. 2021; Świątoniowska-Lonc, et al. 2020; Mussie, Gradmann, and Manyazewal 2020; Saito, et al. 2021). Language is an important factor to establish an effective patient–physician relationship and improve the provision of quality healthcare, especially in the context of refugee healthcare (Świątoniowska-Lonc, et al. 2020; Samkange-Zeeb, et al. 2020). In NGO settings, this can be challenging due to time pressure, human resource shortages, and the increasing number of patients seeking treatment. As a result, informed consent may not always be prioritized due to resource constraints. Although helping the refugees by prioritizing pain alleviation is a beneficial act, the absence of providing patients with all possible treatment options for them to exercise their autonomy is a serious ethical concern warranting much attention. Refugee patients should be given the needed information prior to treatment and assisted to provide informed consent. There should be more efforts to allow and assist refugees to exercise their capacity of action voluntarily (Straehle 2020). The challenges we discussed above in ensuring autonomy in healthcare decision-making among refugees in Germany are also reported in studies in other countries such as Turkey and Australia (Sevimli 2022; Essex 2019). However, and as we established above, the NGO-refugee oral healthcare context is poorly researched and studies mostly report general healthcare for refugees. This indicates the urgent need to further investigate this complexity.

## Conclusion and Recommendations

In this article, we discussed the gaps in oral healthcare services provided for refugees in NGO settings in Germany and how they relate to the principles of justice and autonomy. Oral health problems being one of the most common health conditions among refugees, their limited availability for refugee patients shows a lack of justice in healthcare. Even when oral healthcare services are provided in NGOs, refugee patients are less likely to receive enough information about the treatments and thus, less likely to exercise their autonomy.

Although suggesting a detailed strategy falls outside of the scope of this work, we point out a few brief recommendations which might be helpful for further efforts and are based on our analysis which is, as we established earlier, limited by the availability of adequate evidence on the topic. First, in order to ensure better oral healthcare for refugees in Germany, the gaps in the existing legal conditions for refugees should be addressed. There should be more ways to provide healthcare coverage (including oral health services) regardless of, and without the need to change, the refugee status of asylum seekers. Second, more research and analysis could provide a better understanding of the gaps in providing oral healthcare for refugees in Germany and beyond. Although any type of research could be helpful, we specifically suggest that a study employing an exploratory qualitative approach would be most suitable as the area is under-researched (Mansourian 2008; Gerring, Mahoney, and Elman 2020). More specifically, for example, qualitative interviews with refugees receiving oral healthcare from NGOs, and administrators together with care providers in NGOs providing oral healthcare for refugees could explore the (administrative and clinical) challenges of providing/receiving oral healthcare among refugees.

Third, and lastly, more efforts are needed to implement patient autonomy, for example, a fast communication tool such as “Google Translation” and other similar translation software could be used when informing refugee patients about the treatment course. Digital communication assistance tools are useful and effective in addressing language barriers when working with refugee patients who speak foreign languages (Müller, et al. 2020). This method is fast, reliable, and cost-effective for establishing good communication and the patient–physician relationship. This tool enables the dentist to inform the patient about the whole treatment modalities, benefits, and side effects of the treatment and the medical decision that is going to be taken. This could help the patient understand the treatment procedure and provide informed consent.
